# Application of the oxycodone templated molecular imprinted polymer in adsorption of the drug from human blood plasma as the real biological environment; a joint experimental and density functional theory study

**DOI:** 10.3389/fchem.2022.1045552

**Published:** 2023-01-05

**Authors:** Maryam Khanlari, Bahram Daraei, Leila Torkian, Maryam Shekarchi, Mohammad Reza Manafi

**Affiliations:** ^1^ Department of applied Chemistry, South Tehran Branch, Islamic Azad University, Tehran, Iran; ^2^ Department of Toxicology and pharmacology, School of pharmacy, Shahid Beheshti University of Medical Sciences, Tehran, Iran; ^3^ Research Center of Modeling and Optimization in Science and Engineering, Islamic Azad University, South Tehran Branch, Tehran, Iran; ^4^ Food and Drug Laboratory Research Centre, Food and Drug Organization, MOH&ME, Tehran, Iran

**Keywords:** solid phase extraction, molecular imprinted polymer, blood plasma, density functional theory, drug residue, oxycodone

## Abstract

In this project, we have synthesized and used a molecular imprinted polymer (MIP) for adsorption of oxycodone residue from the biological samples. Indeed, this study aims to develop a suitable method for determination of oxycodone drug residue in the human plasma using the common analysis methods. Therefore, the MIP was used for the solid phase extraction (MIP-SPE) approach in order to collect the oxycodone opioid and to concentrate it in the blood plasma samples. The extraction parameters such as adsorption time, pH, and the amount of sorbent in blood plasma were optimized and the capacity of loading amount (LA) for adsorbing it was determined. Moreover, a high performance liquid chromatography (HPLC)-UV detector method was validated and used for analyzing of the mentioned opioid extracted from plasma. The results showed that the limit of detection (LOD), and the limit of quantization (LOQ) for the developed MIP-SPE method were 1.24 ppb, and 3.76 ppb, respectively. Moreover, both of the MIP-, and non-imprinted polymers (NIP)-drug complexes were designed and were then optimized by the density functional theory (DFT) method. The results showed that the theoretical calculations supported the experimental data, confirming the favorability of adsorption of the drug by MIP compared to NIP.

## Graphical Abstract

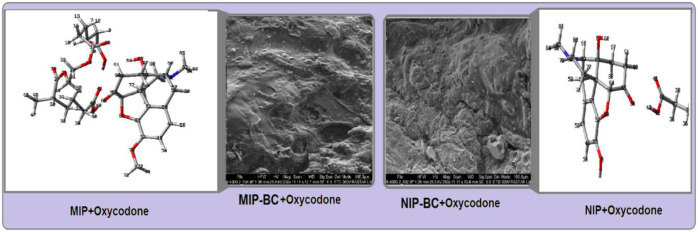

## 1 Introduction

**FIGURE 1:**
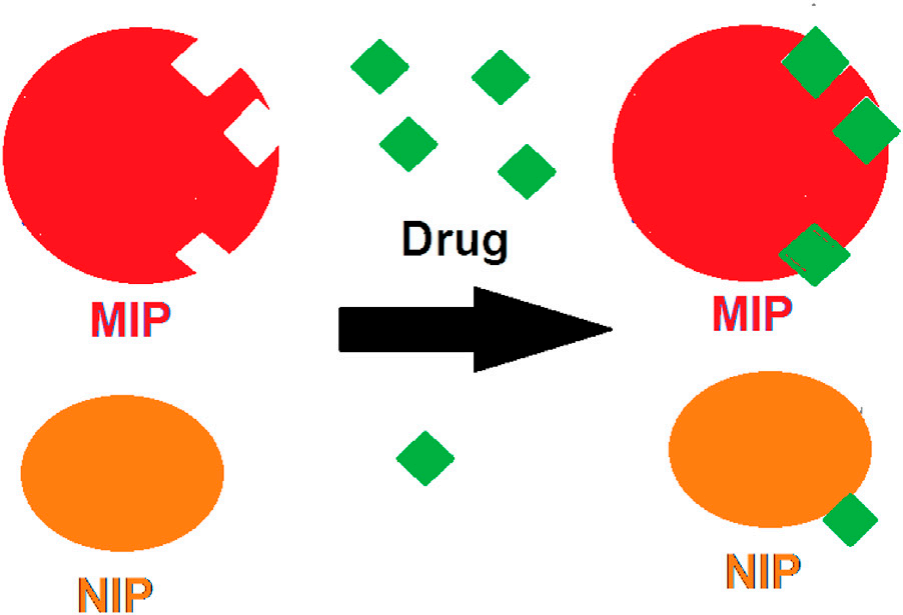
The absorption of the drug molecules by MIP in comparison with its adsorption process by NIP.

The oxycodone opioid (as a six-membered heterocyclic ring) is definitely one of the most important selections in order to relieve severe pains like the cancer ailments. That is, this compound has a wide range of applications in different types of illnesses. Due to this, several formulations of this opioid (like the controlled-release tablets, or liquid crystal forms) have been produced (Ladebo et al., 2020). Indeed, such variation in drug formulation might be due to the importance of the issue of the release control, especially in the case of this opioid. It is more momentous where, in one hand, the bioavailability and the release rate of a pharmaceutical ingredient is high. But, on the other hand, the half-life of that compound and the required dosage for body is low (Pramanik and Thakkar, 2020). Thus, there are a variety of approaches which have been designed to regulate the release rate of drugs like the slow-release tablets (de Salazar et al., 2021), the osmotic pumps (Brunaugh et al., 2019), the sol-gels (Raboh et al., 2021), and the MIPs (Shi et al., 2020). The MIP granules are the achievements of the molecular imprinting technique (MIT) which have been developed as suitable candidates for drug delivery applications in the recent decade (Motaharian et al., 2020). In such models, the MIP polymers are formed in the presence of a molecular template (the selected drug) (Figure 1). Then, by completing the polymerization process, the product is washed for several times to make the MIP free of those template molecules. Thus, the produced MIP is full of free substrates which could be attached to the molecules of the selected drug for further use (Behnia et al., 2022; Hoseini Chehrghani et al., 2022). It would be clear that such valuable composition could be applied as a stationary phase for the chromatography columns packing (Hroboňová and Lomenova, 2018), synthesized sorbents (Hroboňová and Brokešová, 2020), nano actuators (Motaharian et al., 2019), biosensors (Feroz and Vadgama, 2020), and also as nano-scale drug release machines (Wu et al., 2018).

Due to the above mentioned facts, in the recent decade, the MIP products are reported to be applied as suitable candidates for the new generation of the drug delivery systems (DDS). Somehow, by using this technique, physician would be able to make accurate dosages and to regulate the release properties of the drugs (Zhang et al., 2018;Joy et al., 2022) which are the key advantages of this system.

Thus, in the present project, we have applied oxycodone as the molecular template to prepare the MIP granules for further use in adsorption (Mirabi et al., 2016) and pre-concentration (Sharghi et al., 2017; Gupta et al., 2019) of this drug in the human blood plasma. The morphology investigations on the composites were carried out by using the scanning electron microscopy (SEM). Moreover, the drug adsorption kinetics by the MIPs were investigated with the aid of the high performance chromatography (HPLC) (Hossaini et al., 2015; Dadras et al., 2022), (Katakam and Katari, 2021). Indeed, one of the main aims of this project was to evaluate the drug adsorption process from the blood plasma by using the kinetic models and to determine their mechanism of adsorption. Also, one of the other principles of this research was to clarifying this adsorption process in the molecular view. Thus, the adsorption process of oxycodone by both of the MIPs, and NIPs were probed by the density functional theory (DFT) quantum chemical method (Asadi-Ojaee et al., 2019; Padash et al., 2021). The thermodynamic data extracted by the frequencies confirmed the experimental results revealing that MIP could make stronger interactions with the drug compared to that of NIP.

## 2 Experimental

### 2.1 Materials and reagents

All of the solvents and the chemical reagents were analytical grade. The ultra-pure water was obtained by a Milli-Q purification system (Millipore, Bedford, MA, United States). The chemical compounds containing methacrylic acid (MAA), 2,2-azobisissobutyronitrile (AIBN), ethylene glycol dimethacrylate (EGDMA, 98% purity), polycaprolactone-triol (PCL-T), N,N-dimethyl formamide (DMF), and N-Methylmorpholine N-oxide (NMMO) were purchased from Sigma-Aldrich. Moreover, monobasic potassium phosphate (KH_2_PO_4_) and acetonitrile were obtained from the Merck Chemical Company. Also, the oxycodone active pharmaceutical ingredient (API), and the human blood plasma (ABB group) were prepared from commercial sources, and from the Iranian Blood Transfusion Organization (IBTO), respectively.

### 2.2 Instrumentation

The Shimadzu Prominence high performance liquid chromatography (HPLC) instrument (Shimadzu Corporation, Kyoto, Japan) which was equipped with a LC-20AD pump, a DGU-20A degassing agent, a SPD-20A UV-Vis detector, and also a CTO-20A column oven, were applied for all of the following analysis. Moreover, the Lab-Solutions software version 5.51 was aided for the data analysis and subsequent processing. Also, a (250 × 4.6) mm, 5 µm, C18, end-capped liquid chromatography column was used for the assaying analysis of the concentration of oxycodone. In addition, a FEI Quanta 200 scanning electron microscope (Thermo Fisher Scientific, Netherland), a Spectrum 100 FT-IR spectrometer (PerkinElmer Co., Ltd., United States), a CR3i centrifuge (Thermo Fisher Scientific Inc., United States), a CHZ-82 constant temperature water bath oscillator (Fuhua Instrument Co. Lid., China); and also a KQ2200B sonic device with frequency and temperature controller (Kunshan Ultrosonic Instrument Co., Ltd., China) were applied for all of the analysis processes. Finally, a ZRS-8G dissolution tester (Tianda Tianfa Technology Co., Ltd., China) was used for the release examination.

### 2.3 Preparation of MIPs

Both of the MIPs and the NIPs were synthesized by the precipitation polymerization method, with the aid of the UV photo polymerization technique. Somehow, at the first step of the process, 1 mmol of oxycodone was dissolved in 75 ml of DMF as solvent. Then, 4 mmol MAA, 20 mmol of EGDMA, and 50 mmol of AIBN were being added to the solution. Then, it was taken under ultrasonic waves for 10 min. In addition, the solution was purged by the molecular nitrogen atmosphere for about 10 min. Subsequently, the reaction vessel was putted under UV irradiation at 366 nm for 24 h. Finally, the MIP, and NIP were placed to the oven for drying and grinned for further use (Behnia et al., 2022; Hoseini Chehrghani et al., 2022).

The prepared polymer was then powdered by grinding with a suitable mortar. Meanwhile, a reference of NIP was yielded under identical conditions without the presence of oxycodone as the molecular template. The schematic procedure for obtaining the MIP was presented in Figure 1.

### 2.4 Characterization of the MIPs

#### 2.4.1 SEM analysis

The morphologies of the MIP granules were studies with the SEM. Those photographs were obtained with a FEI ESEM QUANTA 200 (MIPs). The surfaces and cross-sections of the loaded as well as un-loaded MIPs and NIPs were made conductive by deposition of a gold layer on the samples into a vacuum chamber. Moreover, the morphologies of the samples were investigated under SEM scanning at the voltage of 25 kV, respectively.

#### 2.4.2 FT-IR analysis

The FT-IR spectra of the MIPs and the NIPs were obtained by FT-IR analyzer. To do so, a certain amount (5 mg) of the MIP and NIP powders and a 100 mg powder of KBr were mixed and grinded well. Then, the mixture was pressed into 1 mm pellets. The prepared samples were measured on a Spectrum 100 FT-IR spectrometer, respectively. The FT-IR spectra of the MIP and NIP were plotted by recording from 4,000 to 400 cm^−1^ with a resolution of 2 cm^−1^ by using a pellet of potassium bromide as the reference.

### 2.5 Data analysis

For studying the oxycodone transportation mechanism by the granules, three adsorption patterns containing the Pseudo-second-order adsorption model, the First order and the Zero order patterns were applied. The kinetic studies were performed by plotting the cumulative values of the drug (in percentage) per time (in hours). The correlation coefficient (r) for each kinetic model was calculated to give the model that was followed.

The zero order model (concentration per time)

$$Q=Q_{0}+K_{0}t$$ (1)

Pseudo-second-order adsorption model (concentration per square root of time);

$$t/Q_{t}=1/K_{2}Q_{e}^{2}+Q_{t}$$ (2)

where, K_2_ (mg min^−1^) is the rate constant of pseudo second order adsorption. From the boundary conditions t = 0 to t = *t* and Q_t_ = 0 to Q_t_ = Q_t_.

First order model (logarithm of the concentration per time);

Log Qt=log⁡QKt (3)

where Q_t_ is the amount of adsorption (mg) per time t (h), Q_0_ is initial amount in donor compartment (µg). Also, K_0_ is zero order constant (µg h^−1^), K_1_ is first order constant (µg h^−1^), and K_2_ is Pseudo-second-order adsorption rate constant (µg h^1/2^). The correlation coefficient (r) for each kinetic model was calculated to find the most reliable model.

$$Qt=\left(C_{0}-C_{t}\right)V/m$$ (4)

$$\alpha =Q_{MIP}/Q_{NIP}$$ (5)

where Q_t_ (μmol/g) is the adsorption capacity at different times, C_0_ (mmol/L) is the initial concentration of quetiapine drug, Ct (mmol/L) is the concentration of the drug at the time t, V (in Liter) is the volume of the initial quetiapine drug solution, and m is the mass of NIP and MIP. Finally, α is the selectivity factor both for NIP and MIP, Q_NIP_ (μmol/g) is the adsorption capacity of the NIP, and Q_MIP_ (μmol/g) is the adsorption capacity for MIP.

#### 2.5.1 Adsorption experiments

Firstly, the standard solutions of oxycodone drug (.02, .1, .2, .4, 1, and 2 μg ml^−1^) were obtained, accordingly. Secondly, 50 µL of each standard solution was added to 1 ml of the drug-free blood plasma to give 1, 5, 10, 20, 50, and 100 ng ml^−1^, respectively. Then, each of the prepared samples was introduced to a 1 ml of 15% of perchloric acid and vortexed for 1 min. Next, each mixture was being centrifuged for 5 min at 4,000 rpm and the supernatant was added to .5 ml of a .5 M of sodium hydroxide solution. Subsequently, the volume of the solution was increased to 10 ml by pH = 7 Sodium Phosphate Dibasic buffer solution, and a 10 mg of MIP was added to that. The prepared mixture was vortexed for 5 min and centrifuged for about 5 min at 4,000 rpm until the supernatant was separated. It was then introduced to 5 ml of pH = 2 buffer solution for 20 min. Finally, the last solution was taken under centrifuge and the supernatant was given to HPLC-UV system to determine the concentration of the adsorbed/released drug. Same analyzes were performed for NIP as reference.

### 2.6 High performance liquid chromatography method

The determination of oxycodone concentration and release was performed by a Shimadzu HPLC system occupied with a UV/VIS detector set to 210 nm. The separation was performed by an end capped ortho diesel silane (ODS) C18 (4.6 mm, 250 mm, 5 μm) liquid chromatography column. The injection volume was accurately 10 μl, the flow rate of the elision was 1 ml/min and the column temperature was being fixed at 25°C by using the column oven. Also, an isocratic method was applied for the elusion and the mobile-phase was ACN/buffer solution with a 40/60, v/v percentage. Where, the buffer was made by dissolving the 10 mM of monobasic potassium phosphate in 1 L of purified water set at pH of 6.4 by using phosphoric acid.

### 2.7 Quantum chemical calculations

The isolated structures of the oxycodone drug and the polymer of MAA- EGDMA (both in liner, and complex forms) were drawn as input files and were then optimized to give the most stable energy minima. Then, the complex systems of the polymer in each form with the drug were subsequently designed and putted under the related calculations. The Gaussian 03 quantum chemical software was applied to perform the desired calculations (Frisch et al., 2003). Also, the thermodynamic parameters were extracted accordingly. Studies on all stationary points in addition to the other required calculations were performed by applying the B3LYP/6–31g(d) level of theory (Latypov et al., 2015; Zeroual et al., 2015) which has been shown to be suitable for these type of investigations (Siadati et al., 2016; Pakravan and Siadati, 2017; Vessally et al., 2017; Mohtat et al., 2018).

Also, the key parameters for the reactivity descriptors (Siadati and Alinezhad, 2015; Frau and Glossman-Mitnik, 2018; Choudhary et al., 2019; Üstün et al., 2019) have been calculated by the following formula:

Global hardness:

$$\eta =\left(I-A\right)/2$$ (6)

where; I (Ionization potential) is the negative of the energy level of HOMO (-*E*_HOMO_), A (Electron affinity) is the negative of the energy level of LUMO (-*E*_LUMO_).

Softness:

S=1/2η (7)

Electronegativity:

$$X=\left(I+A\right)/2$$ (8)

Electrophilicity index:

$$\omega =\mu^{2}/2\eta$$ (9)

where, *µ* is the chemical potential:

$$\mu =\left(I+A\right)/2$$ (10)

## 3 Results and discussion

### 3.1 Structural characterization of the MIP

#### 3.1.1 FT-IR spectroscopic analysis

**FIGURE 2:**
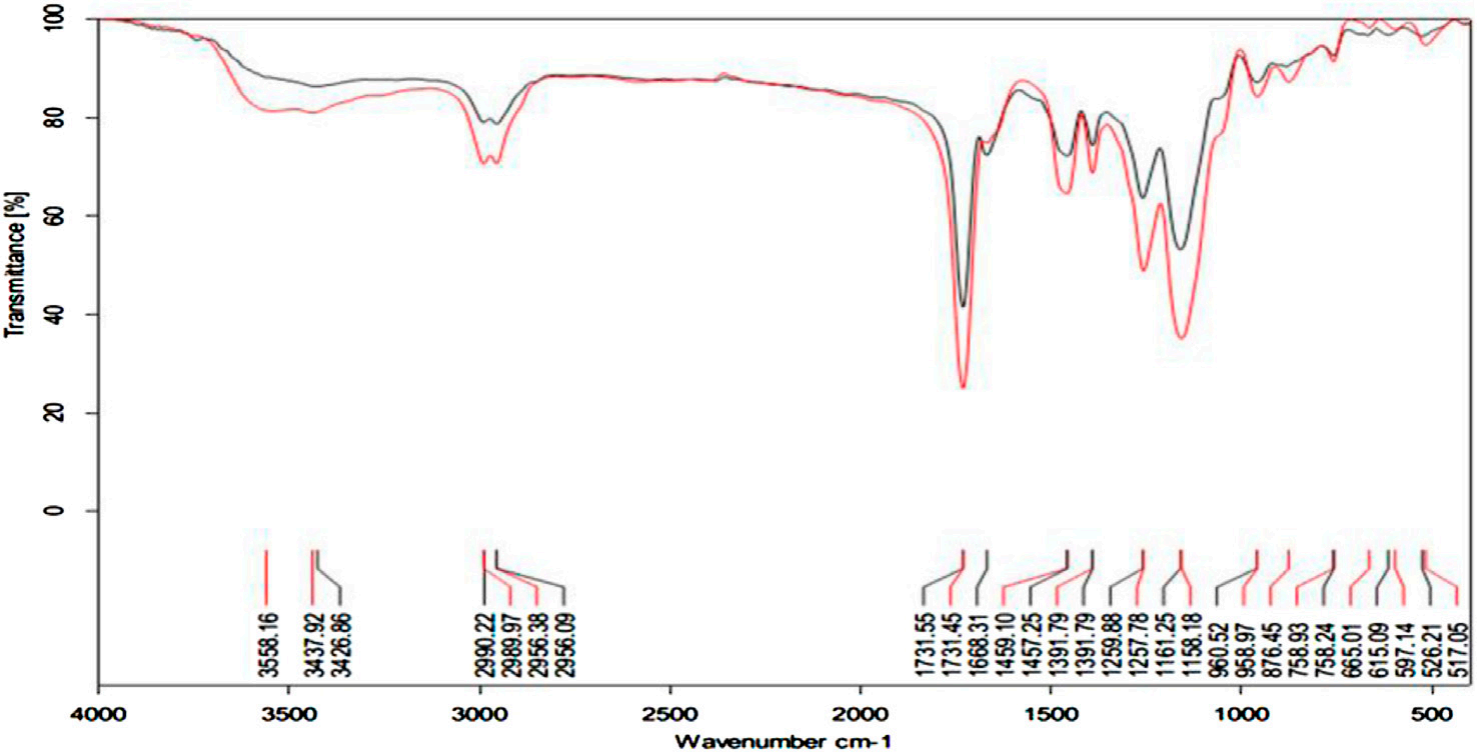
FT-IR spectra for the unloaded MIP (black line), and the oxycodone loaded MIP (red line).

The FT-IR spectra of unloaded MIP, and the oxycodone loaded MIP, were shown in Figure 2. The unloaded MIP had strong absorption peaks at *ν*C-O (stretching) = 1,257 cm^−1^, *ν*C = O (stretching) = 1731.45 cm^−1^, and *ν*O-H (stretching) = 3,437 cm^−1^, due to introduction of OH, as well as EGDMA (cross-linking agent), and carboxyl groups from the MAA (functional monomer).

By comparison, after loading the oxycodone molecule on the MIP, the absorption peaks of C=O, O-H, and C-O peaks, shifted to 1731.55 cm^−1^, 1,259 cm^−1^, and 3,426 cm^−1^, respectively. Moreover, the absorption peak of the O-H bond was increased and strengthened at 3,437 cm^−1^ in the drug loaded MIP, revealing about the formation of a hydrogen bond between MIP and the template molecule. Moreover, appearance of the wide peak in 3,558.16 cm^−1^, in the drug loaded MIP (Red line), compared to the drug free MIP (Black line), might indicate the formation of hydrogen bonds in this complex from.

#### 3.1.2 Morphological structure analysis

**FIGURE 3:**
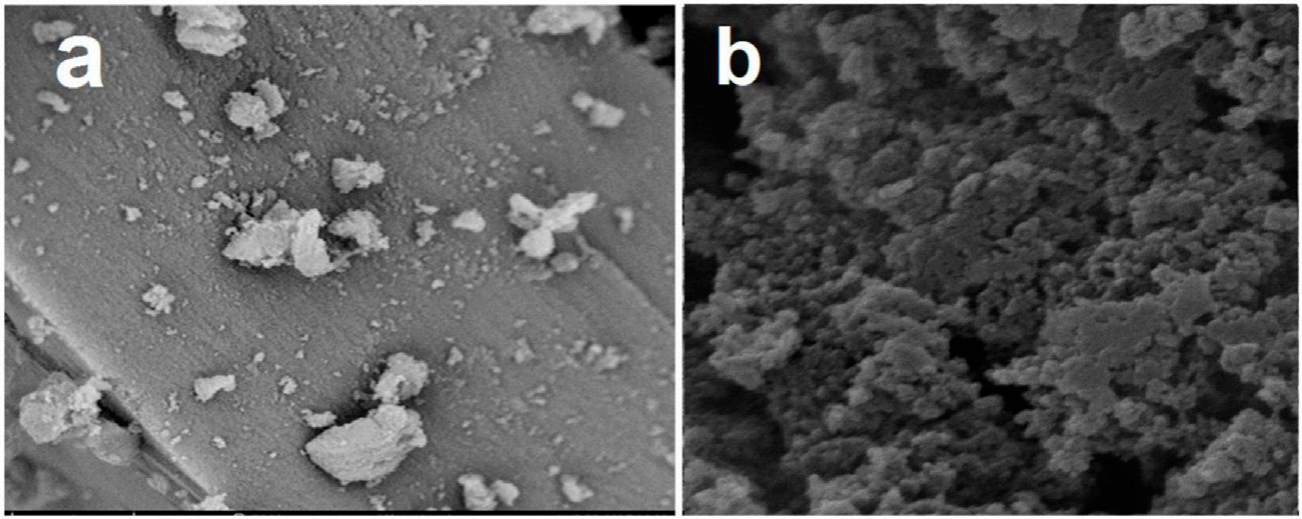
SEM images of the surface of MIP **(A)**, and NIP **(B)**.

As presented in Figure 3, the surfaces of the MIP, and NIP particles have been monitored by using the SEM photographs. These figures show that both MIP and NIP particles have spherical and uniform morphologies. In fact, the SEM images indicate the uniform and regular texture of the nanoparticles. Also, these pictures confirm that both MIPs and NIPs, are in nano scale, due to their diameters. Moreover, there is not a significant difference in view of the morphology between the NIP, and MIP particles. Therefore, loading the oxycodone on the polymer or using this drug as the molecular template in the synthesis of MIP has not changed the surface morphology. Moreover, oligomerization of particles has been observed in some areas of the images. Such happening might be due to the increase in tendency for oligomerization and surface reactivity, in nano scale processing.

#### 3.1.3 Limit of detection and limit of quantification

The results of the work indicate that the limit of detection (LOD) and limit of quantification (LOQ) for this drug extraction-detection procedure are 1.24 ppb, and 3.76 ppb, respectively, which show the accuracy and applicability of the method for determination of the bioavailability of the oxycodone drug in the human plasma.

### 3.2 The adsorption experiments

#### 3.2.1 Optimization of the pH of adsorption

**FIGURE 4:**
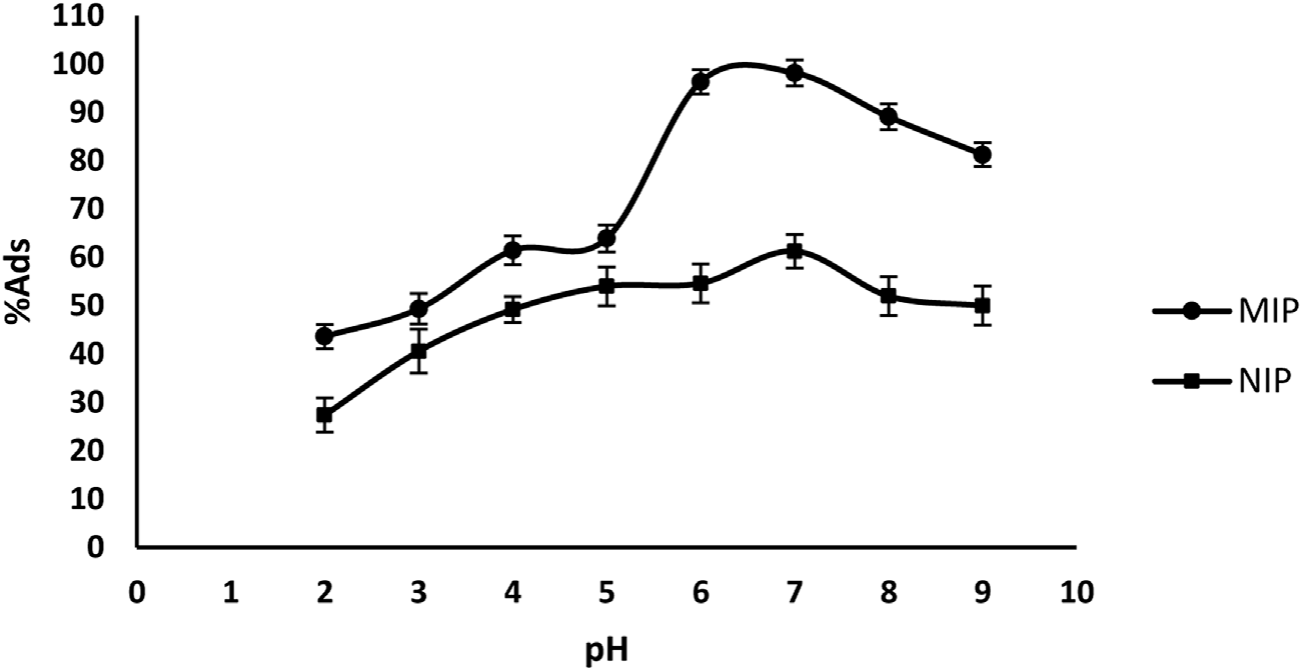
The adsorption of oxycodone drug by MIP and NIP in different pHs.

The different ratios of MIP and NIP were used as the sorbents. The oxycodone adsorption from the MIP and NIP was analyzed in different pHs. The results of Figure 4 shows that in one hand the adsorption of the drug by MIP is clearly higher than that of NIP; and on the other hand, the maximum of adsorption occurs in a pH range from 6.5-7.5 (the pH of the human plasma is about 7.4). Thus, more studies were set on such pH.

#### 3.2.2 Determination of the kinetics of adsorption

**FIGURE 5:**
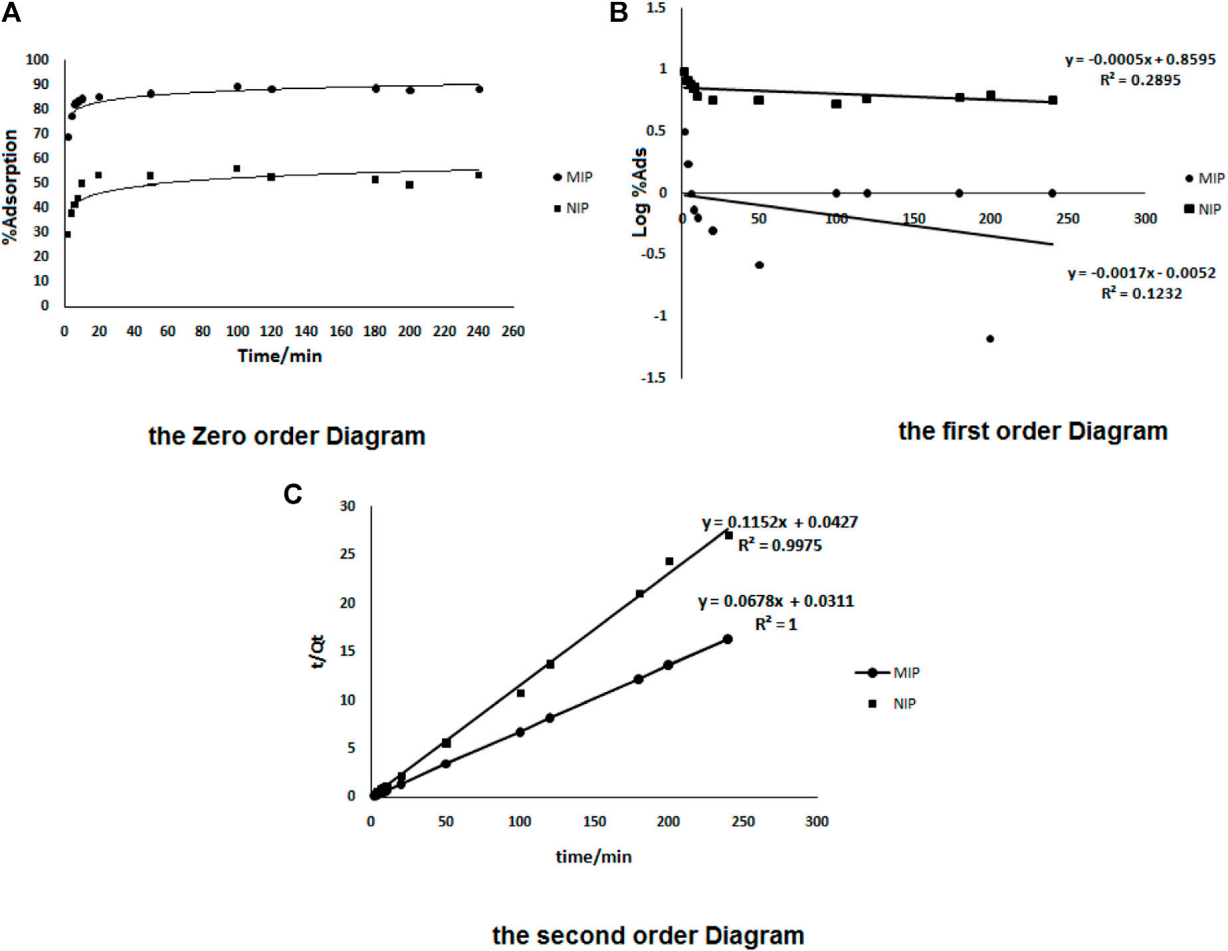
The zero order **(A)**, the first order **(B)**, and the second order **(C)** kinetics models for adsorption of oxycodone by MIP and also by NIP.

The different kinetic models were drawn to give the most reliable kinetic model. As shown in Figure 5, the most linearity was observed in the case of the second order equation plot (part c). It shows that the adsorption of the drug from the solution as a square root of time dependent process can be explained by the physicochemical properties of oxycodone as a polar drug. Such forms could be able to enhance the drug adsorption from the solutions (like blood plasma) as well as to control the drug release. In order to mimic the real conditions of the human body, and an accurate investigation on the release behavior of oxycodone in the drug-loaded MIPs, the absorption experiments were performed in the pH = 7.4 (which is the pH of the human blood serum; as well as the pH optimization results of the drug adsorption section) under continuous stirring at 37 ± 0.5°C (the human body’s normal temperature).

### 3.3 Adsorption of oxycodone from the blood plasma by MIP

Table 1 represents the concentrations of the oxycodone drug which has been absorbed by MIP and by NIP nanoparticles from the different concentrations of the standard solution prepared from blood plasma. First observable outcome of the data is the considerably higher ability of the MIP compared to NIP in adsorption of the drug. Somehow, at the least concentration (10 μg ml^−1^), the percentage of adsorption of the drug by MIP is 87.20% which is significantly higher than that of NIP (45.46%). Moreover, by increasing the concentration of the drug in the plasma, the adsorption percentage slightly increases; somehow, the maximum of the adsorption for MIP (90.91%) occurs in 70 μg ml^−1^. While, at this concentration (70 μg ml^−1^), the adsorption percentage of NIP is only 58.24%. Also, in higher concentrations, the amounts of this value slightly decrease.

**TABLE 1:** The amounts of oxycodone compound, adsorbed by MIP, and NIP in different concentrations of the blood plasma solution. q_e_ is the concenteration of the drug at equlibrium.

Concentration (µg ml−1)-NIP	Adsorption (%)- NIP	qe (mg/g)- NIP	qe (mg/g)-MIP	Concentration (µg ml−1)- MIP	Adsorption (%)- MIP
10	45.46	1.51	2.91	10	87.20
20	47.42	3.16	5.67	20	87.43
40	52.58	7.01	11.68	40	87.57
50	57.05	9.51	14.63	50	87.80
60	57.68	10.94	17.24	60	88.22
70	58.24	13.59	21.21	70	90.91
80	56.29	15.01	22.78	80	85.41
90	55.82	15.85	25.73	90	85.78
100	54.70	19.57	27.24	100	81.71
120	53.92	21.57	32.12	120	80.27

On the other hand, the adsorption of the drug residue from the blood plasma shows that at the lowest time of adsorption (about 2 min), the MIP could adsorb 69.3% of the oxycodone from the blood plasma; while the NIP could only adsorb 29.6% of the compound. By increasing the adsorption time from 2 min to 100 min, the adsorption percentage increases up to 89.2% and 55.8% for MIP, and NIP, respectively (Table 2). However, after 100 min, the adsorption time decreases. It also, indicates the favorability of MIP compared to NIP in adsorbing of oxycodone from the blood plasma. Thus, both the results of the q_t_, and q_e_ indicate that there is a logical relation between the adsorption of the drug by NIP and by NIP. Somehow, when adsorbing the target drug by MIP, the outcome and the rate of the process are significantly higher than that of NIP. Moreover, the results show that the synthesized MIP (and NIP as reference) could be used in the human blood plasma.

**TABLE 2:** The amounts of oxycodone drug, taken from the suppernatants of the plasma the MIP and NIP. q_t_ is the concenteration of the drug at time t.

Time (min)	Adsorption (%)-NIP	qt (mg/g)- NIP	qt (mg/g)-MIP	Adsorption (%)-MIP
2	29.6	4.93	11.56	69.3
4	38.2	6.38	12.93	77.6
6	41.6	6.93	13.72	82.3
8	44.2	7.37	13.96	83.8
10	50.3	8.38	14.06	84.4
20	53.3	8.88	14.20	85.2
50	53.1	8.85	14.44	86.6
100	55.8	9.30	14.87	89.2
120	52.4	8.74	14.72	88.3
180	51.5	8.58	14.76	88.6
200	49.4	8.23	14.63	87.8
240	53.4	8.90	14.70	88.2

### 3.4 Theoretical results

**FIGURE 6:**
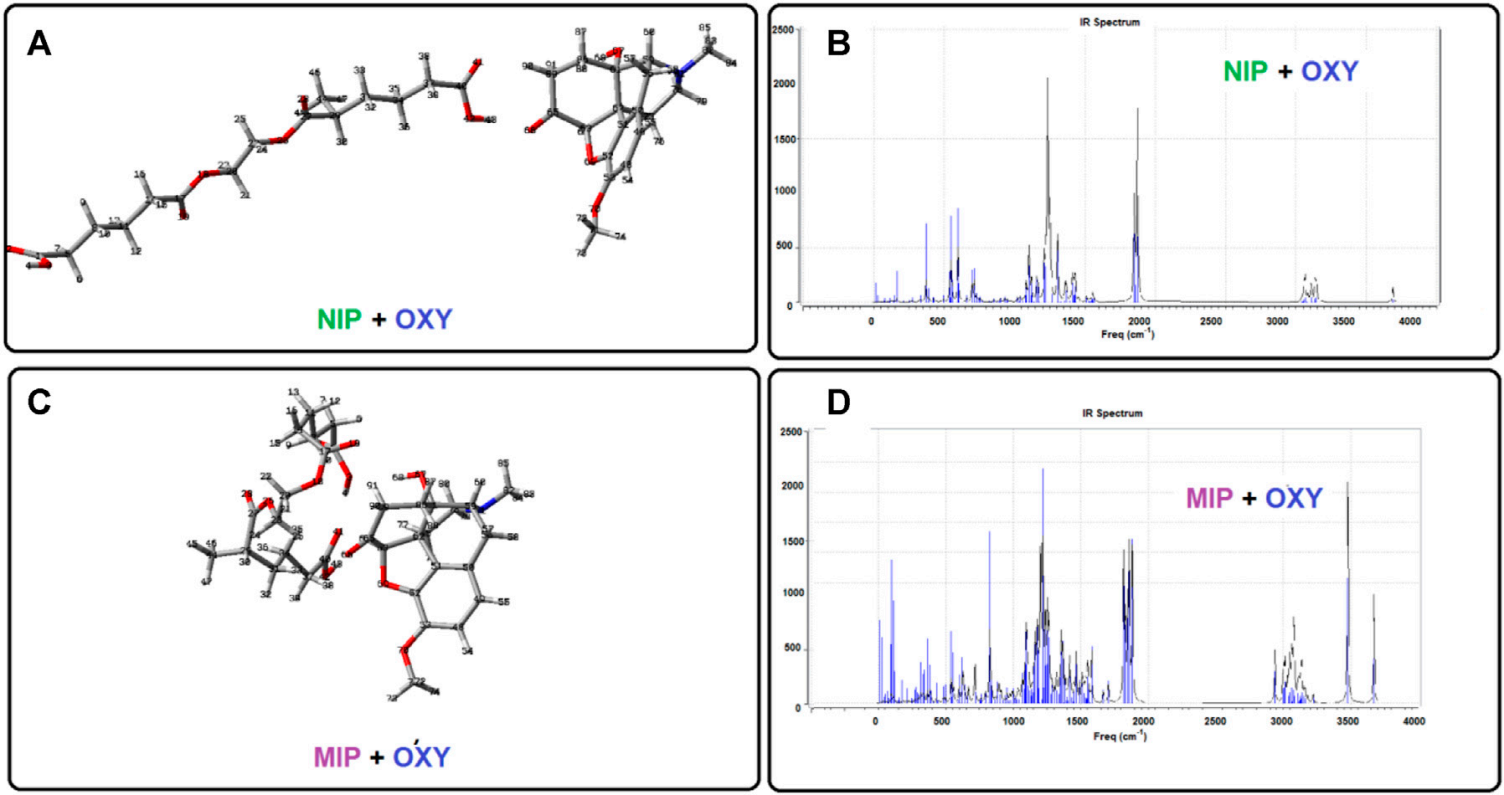
The optimized structures as well as their calculated IR spectrums obtained by the DFT method.

As mentioned above, some of the quantum chemical calculations have been performed to give a better view in the case of the interactions between the host (nano polymer) and the guest (oxycodone drug). Using such studies, we are able to investigate the molecular and atomic behaviors of the chemical species and sub-molecular fragments. To do this, we have designed each of the separated species containing the oxycodone drug, a complex molecular segment as MIP (simulating the imprinted polymer), and a liner molecular fragment as NIP. Then, the designed geometries were made as input files and putted under quantum chemical calculations to give more stable states as output files (Figure 6). In addition, the optimized structure of oxycodone was placed near to NIP, and also near to MIP to give the NIP-OXY, and MIP-OXY systems, respectively. Then, the energy saddles of the best geometrical places were found, and the theoretical level of calculation were evaluated to the B3LYP/6-31G(d).

As shown in Figure 6, there are two sharp peaks between 3,400 cm^−1^ and 3,800 cm^−1^ in the calculated IR spectrum of MIP + oxycodone (part d) systems which have been disappeared in the IR spectrum of the NIP + drug (part b). It would be due to the strong hydrogen bandings in the MIP + drug optimized structure (part c) compared to the optimized structure of NIP + drug (part a). This result is in agreement with the experimental FT-IR in appearance of the wide peak in 3,558.16 cm^−1^, in the drug loaded MIP (Red line), compared to the drug free MIP (Black line), might indicate the formation of hydrogen bonds in this complex from.

Obviously, the DFT approach has been applied as an efficient method for calculation of the chemical reactivity and the substrate selectivity in popular qualitative chemical concepts such as electronegativity (χ), electrophilicity index (ω), chemical potential (μ), softness(S), and chemical hardness (η). Moreover, using the electron affinity (A), Koopmans’theorem ionization potential (I), softness(S), hardness (η), and electronegativity(χ) which are depended on the energy of the HOMO and the LUMO, some of the physiochemical behaviors of the system would be predicted. The data presented in Table 3, indicate the reactivity parameters for oxycodone as well as MIP, and NIP compounds. The result reveals that the HOMO–LUMO energy gap (E_g_) of oxycodone is about −.073 a.u. which is relatively low.

**TABLE 3:** The reactivity descriptors of the oxycodone opioid.

Reactivity descriptor	Reactivity descriptor Energy (a.u.)	Reactivity descriptor	Reactivity descriptor Energy (a.u.)
Electron affinity	0.199	Electronegativity	0.236
Ionization potential	0.272	Electrophilicity index	0.750
Global hardness	0.037	HOMO energy	−0.272
Softness	13.513	LUMO energy	−0.199

Thus, it could reveal about its polarizable nature (due to the low FMO gap, the low kinetic stability, and the high chemical reactivity). In addition, low ionization energy (I) about .272 a.u. Of oxycodone reveals that this compound is highly reactive. Also, the softness parameter was used to measure the extent of chemical reactivity and it is the measure of the ability of an atom or a group of atoms to absorb electrons. Therefore, the softness (13.513 a.u.) of the mentioned drug indicates that this compound could be soft enough to receive electron density from different types of compounds (Table 3).

**FIGURE 7:**
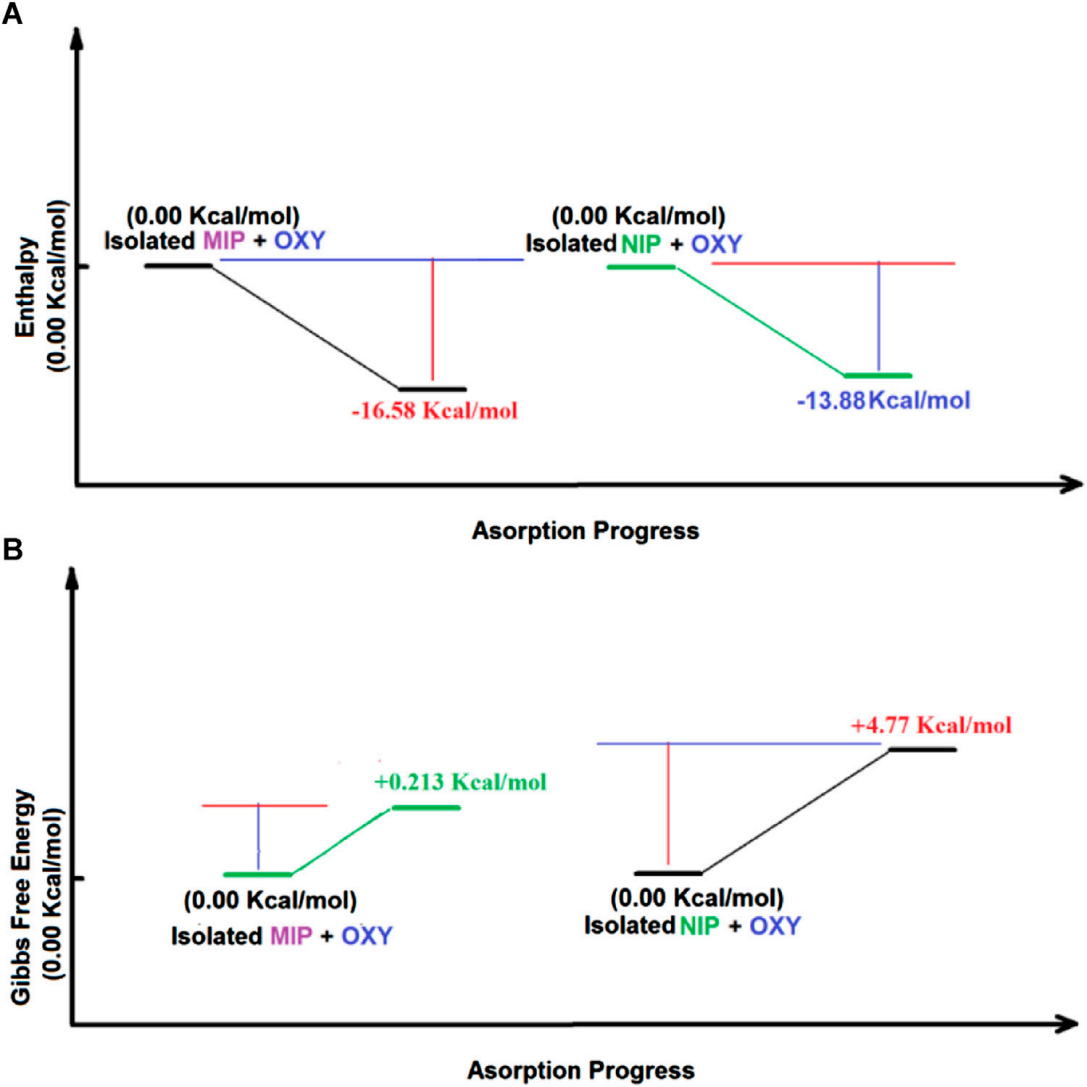
The Enthalpy **(A)** as well as the Gibbs free energy **(B)** changes during the adsorption of oxycodone drug obtained by DFT method.

The thermodynamic studies show that Δ*H*_ads_, Δ*G*_ads_ for adsorption of oxycodone by MIP and NIP, are −13.9 kcal mol^−1^, −16.6 kcal mol^−1^, 4.8 kcal mol^−1^, and .2 kcal mol^−1^, respectively. Thus, the values of ΔΔ*H*_ads_, and ΔΔ*G*_ads_ for the adsorption of the drug by MIP, and NIP are −2.7 kcal mol^−1^, and 4.6 kcal mol^−1^, respectively, which show a high priority of MIP compared to NIP in adsorption of oxycodone drug. Moreover, the ΔS_ads_ for MIP and NIP are −56.320 Cal mol^−1^ K^−1^, and -62.555 Cal mol^−1^ K^−1^, respectively. It indicates that the adsorption of oxycodone by MIP is more preferred than that of NIP in view of entropy. Therefore, the thermodynamic studies by the DFT calculations clearly confirm the experimental results revealing about the suitability of MIP compared to NIP for adsorption of oxycodone drug (Table 4; Figure 7).

**TABLE 4:** The key thermodynamic as well as UV-visible adsorption parameters containing *H* (Hartree/particle), *G* (Hartree/particle), *S* (Cal mol^−1^ K^−1^), λ_max_ (nm) ΔH (kCal mol^−1^), ΔG (kCal mol ^−1^), ΔS (Cal mol^−1^ K^−1^) for each considered species.

Species	H	G	S	ΔH	ΔG	ΔS
Oxy	−1,053.447150	−1,053.508142	128.369	—	—	—
NIP	−1,187.104487	−1,187.174046	146.398	—	—	—
MIP	−1,187.088745	−1,187.162562	155.362	—	—	—
Oxy-NIP	−2,240.573758	−2,240.674587	212.212	−13.881	4.770	−62.555
Oxy-MIP	−2,240.562314	−2,240.670364	227.411	−16.578	0.213	−56.320

## 4 Conclusion

In conclusion, a MIP was synthesized for adsorption of oxycodone from the human blood plasma samples. The results of the validation studies (by HPLC-UV system) in the human plasma as biosamples showed that the adsorption of the oxycodone drug loaded on MIP was higher than that of NIP. Moreover, the results indicated that the release kinetics was fitted with the Pseudo-second-order adsorption model. Moreover, in order to find the reason of the priority of the MIP in adsorbing the oxycodone drug from the blood samples, we have used the theoretical quantum chemical methods. To do that, both the MIP, and the NIP host-drug systems were studied by the DFT approach. The thermodynamic calculations showed that Δ*H*_ads_, Δ*G*_ads_ for adsorption of oxycodone by MIP and NIP, are −13.9 kcal mol^−1^, −16.6 kcal mol^−1^, 4.8 kcal mol^−1^, and .2 kcal mol^−1^, respectively. Therefore, the values of ΔΔ*H*_ads_, and ΔΔ*G*_ads_ for the adsorption of the drug by MIP, and NIP were -2.7 kcal mol^−1^, and 4.6 kcal mol^−1^, respectively. It indicated a high priority of MIP compared to NIP in adsorption of oxycodone drug. Moreover, the ΔS_ads_ for MIP and NIP are −56.320 Cal mol^−1^ K^−1^, and −62.555 Cal mol^−1^ K^−1^, respectively. It showed that the adsorption of oxycodone by MIP is more preferred than that of NIP in view of entropy. Therefore, the thermodynamic studies by the DFT calculations clearly confirm the experimental results revealing about the suitability of MIP compared to NIP for adsorption of oxycodone drug. Finally, the data show that the LOD and LOQ for this drug extraction-detection procedure are 1.24 ppb, and 3.76 ppb, respectively. It might be a proof that shows this method is accurate and applicable for determination of the bioavailability of the oxycodone drug in the human plasma.

## Data Availability

The original contributions presented in the study are included in the article/Supplementary Material, further inquiries can be directed to the corresponding authors.
